# Surgery

**Published:** 1906-02-10

**Authors:** 


### SURGERY.

**Dislocations and Fractures.**—A remarkable case of forward dislocation of the atlas on the axis is related by Kelly.[1] A man of forty-seven fell when intoxicated and struck his head on a sharp corner. His head was fixed in a position of flexion and the spine of the axis was abnormally prominent. An *x*-ray photograph proved the diagnosis of fracture dislocation to be correct. The odontoid process had been broken off the axis and carried forward by the displaced atlas. As a result of this no sensory or motor nerve paralysis or any grave symptoms were produced. It is probable that when traumatic dislocation of the atlas forwards on the axis takes place without causing immediate death, that the odontoid process is always broken off. Otherwise, if by a slipping under the transverse ligament or by the rupture of the latter, the odontoid peg remains in place, nothing can prevent the medulla being crushed between it and the posterior arch of the atlas, with fatal results. The patient made a good recovery, no treatment being used except the wearing of a felt collar. Ware [2] refers to the difficulty of applying satisfactory splints to the fractured femur of young children. He speaks most highly of an apparatus designed by Arsdale and which he has used in forty cases with excellent results in all. It consists of a triangular frame made of cardboard, one side is made to fit from the axilla to the groin, another from the groin to the knee, these two sides lying at right angles to each other. The base of the triangle connects the axilla to the knee. The triangle is bandaged to the trunk and thigh with starch bandages, and fixes the injured limb in a position of abduction and rotation outwards, the leg being left to hang loose. This position places the extensor muscles of the leg and the flexors of the hip at rest and so prevents angular deformity. Also the abducted position makes it easy to keep the child clean. Wharton,[3] in relating three cases of fracture of the head of the tibia, draws attention to the following important points. The injury is caused by indirect violence in which the body is rotated on the leg when the latter strikes the ground. The external tuberosity is usually fractured from the head of the bone and the line of fracture enters the joint. This fact, together with the occasional involvement of the external popliteal nerve, causes great pain. The fracture requires a long time for complete union, often as long as four months. And the functional results are even then not very perfect owing to damage of the joint structures. Davis [4] reports a case of a man aged forty-seven who by falling on to the right hip from a height of eight feet had fractured the neck of the femur, the line of fracture was partly intra and partly extra capsular. There was one inch shortening. On the sixth day after the injury an incision was made over the outer side of the trochanter and a 3-inch screw driven into the head of the femur through the great trochanter, traction being made on the leg during this procedure. The functional result was very good, but the screw had to be removed eight weeks after its insertion because of a sinus caused by it. There can be no doubt that in middle-aged patients with fracture of the neck of the femur in which there is much shortening and a difficulty in efficient replacement, that this method will be of great value. It is much better to apply it primarily—*i.e.* within ten days of the accident, than to wait to see what conservative treatment can effect. If the latter plan be adopted, then by the time the operation is attempted the bone will have undergone absorption and the muscles and ligaments will be contracted. As regards the relation between the time of wiring fractures and their healing, Flint [5] makes a very interesting observation. In writing on the subject of infection of the knee-joint he analyses over 100 cases of fractured patella which had been wired. In those which were operated on after the fifth day only 1.2 per cent. became infected, whereas of those done before the fifth day no less than 10.5 per cent. were infected. This clearly shows that in all operations on fractures it is much better to wait until the end of the first week after the injury, because during this time the injured tissues recover their vitality and develop a protective phagocytosis which makes the healing process much more able to resist microbic invasion.

[1] Annals of Surg., Aug. 1905. [2] Ibid. [3] Ibid., Nov. 1905.
[4] Trans. of Philadelphia Academy of Surg., 1905, p. 282.
[5] Annals of Surg., Oct. 1905.

